# Management of painful clitoral neuroma after female genital mutilation/cutting

**DOI:** 10.1186/s12978-017-0288-3

**Published:** 2017-02-08

**Authors:** Jasmine Abdulcadir, Jean-Christophe Tille, Patrick Petignat

**Affiliations:** 1 Department of Obstetrics and Gynecology, Geneva University Hospitals, 30 Bld de la Cluse, 30 Bld de la Cluse, 1211 Geneva, Switzerland; 2 Faculty of Medicine, University of Geneva, Rue Michel Servet 1, 1205 Geneva, Switzerland; 3 Division of Clinical Pathology, Geneva University Hospitals, Rue Michel Servet 1, 1205 Geneva, Switzerland

**Keywords:** Female genital mutilation, Female genital cutting, FGM, FGC, FGM/C, Clitoris, Clitoral reconstruction, Neuroma

## Abstract

**Background:**

Traumatic neuromas are the result of regenerative disorganized proliferation of the proximal portion of lesioned nerves. They can exist in any anatomical site and are responsible for neuropathic pain. Post-traumatic neuromas of the clitoris have been described as an uncommon consequence of female genital mutilation/cutting (FGM/C). FGM/C involves partial or total removal of the female genital organs for non-therapeutic reasons. It can involve cutting of the clitoris and can cause psychological, sexual, and physical complications. We aimed to evaluate the symptoms and management of women presenting with a clitoral neuroma after female genital mutilation/cutting (FGM/C).

**Methods:**

We identified women who attended our specialized clinic for women with FGM/C who were diagnosed with a traumatic neuroma of the clitoris between April 1, 2010 and June 30, 2016. We reviewed their medical files and collected socio-demographic, clinical, surgical, and histopathological information.

**Results:**

Seven women were diagnosed with clitoral neuroma. Six attended our clinic to undergo clitoral reconstruction, and three of these suffered from clitoral pain. The peri-clitoral fibrosis was removed during clitoral reconstruction, which revealed neuroma of the clitoris in all six subjects. Pain was ameliorated after surgery.

The seventh woman presented with a visible and palpable painful clitoral mass diagnosed as a neuroma. Excision of the mass ameliorated the pain.

Sexual function improved in five women. One was not sexually active, and one had not yet resumed sex.

**Conclusion:**

Post-traumatic clitoral neuroma can be a consequence of FGM/C. It can cause clitoral pain or be asymptomatic. In the case of pain symptoms, effective treatment is neuroma surgical excision, which can be performed during clitoral reconstruction. Surgery should be considered as part of multidisciplinary care. The efficacy of neuroma excision alone or during clitoral reconstruction to treat clitoral pain should be further assessed among symptomatic women.

## Plain english summary

Post-traumatic neuromas are benign tumors that arise after surgery or injury to a nerve. The neuromas are the result of disorganized growth and can happen anywhere in the body. Genital traumatic neuromas are rare; few case reports are available in the literature, and these are mainly post-circumcision.

Female genital mutilation/cutting (FGM/C) involves partial or total removal of the female genital organs for non-therapeutic reasons. It can involve cutting of the clitoris and can cause psychological, sexual, and physical complications. Post-traumatic neuromas of the clitoris have been described as an uncommon consequence of FGM/C but very little evidence exists on this subject. In this paper we study the symptoms and management of seven women presenting with a neuroma of the clitoris after FGM/C and discuss how clitoral reconstruction can treat clitoral pain symptoms. Clitoral reconstruction is a surgical technique that consists of removing the scar from FGM/C around the clitoris and exposing the remaining clitoris buried under the scar in a more accessible position. This surgery is relatively recent and conclusive evidence on its safety and effectiveness is lacking. Our findings show that post-traumatic clitoral neuroma can be a consequence of FGM/C. It can cause clitoral pain or be asymptomatic. In the case of pain symptoms, effective treatment is surgical removal of neuroma, which can be performed during clitoral reconstruction. This surgery should be associated to comprehensive psychosexual care. In addition, its results in treating clitoral pain of women should be further studied.

## Background

Post-traumatic neuromas are benign tumors that arise after resection or injury to a nerve. The neuromas are the result of regenerative disorganized proliferation of the proximal portion of the lesioned nerve, and this can exist at any anatomical site [1]. Genital traumatic neuromas are rare; only one study with 17 cases of traumatic neuromas of the penis and several penile and clitoral case reports are available in the literature, and these are mainly post-circumcision [2]. Female genital mutilation/cutting (FGM/C) involves partial or total removal of the female genital organs for non-therapeutic reasons. It can involve cutting of the clitoris and can cause psychological, sexual, and physical complications [3]. Post-traumatic neuromas of the clitoris have been described as an uncommon consequence of FGM/C [3]. Three cases have been reported in the literature [4–6], and these reports discuss cases of women with FGM/C, who presented with a painful clitoral mass that was successfully treated with surgical removal. In each of these cases, results showed that the mass was a post-traumatic neuroma of the clitoris [4–6]. The authors of these studies hypothesized that clitoral neuromas after FGM/C are likely a common condition that is under-reported and under-diagnosed [4, 5]. Indeed, we recently published a two-case study on clitoral reconstruction, where we found that the peri-clitoral fibrosis removed during surgery revealed a post-traumatic neuroma of the clitoris in both subjects [7]. However, no clitoral painful mass was present upon examination of the two women, and only one of them suffered from chronic clitoral pain [7].

Clitoral reconstruction is a surgical technique that consists of removing the peri-clitoral scar from FGM/C and re-exposing the clitoral stump as a neo-glans after sectioning the suspensory ligament of the clitoris. This has been shown to improve clitoral pain, sexual pleasure, and body image [8]. Because of that, this surgery is currently indicated/performed to treat clitoral pain, sexual dysfunction or improve body image [8]. However, there is very little evidence available about its safety and efficacy [9]. The mechanisms for how clitoral reconstruction results in decreased pain symptoms remain poorly understood.

Our aim was to retrospectively evaluate the symptoms and management of women presenting with a neuroma of the clitoris after FGM/C and to discuss how clitoral reconstruction can treat clitoral pain symptoms.

## Methods

We identified all clitoral neuroma cases of women who attended our specialized clinic for women with FGM/C at the Department of Gynecology of the Geneva University Hospitals between April 1, 2010 and June 30, 2016. Since April 2010, our clinic has seen around 15 women per month for a variety of reasons, including surgical treatments such as defibulation and clitoral reconstruction. Clitoral reconstruction, according to the technique described by Foldès [8], has been available since January 2013. Foldès’ technique consists in resecting the FGM/C cutaneous scar covering the clitoral stump; dissecting the clitoris up to its elbow and removing the subcutaneous periclitoral fibrosis. The suspensor ligament of the clitoris is then sectioned avoiding any lesions to the neurovascular pedicle of the clitoris. Once the body of the clitoris is released, this is fixed to the two bulbocavernous muscles to create a healthy and more accessible neoglans [8]. Excision of eventual clitoral neuromas within the FGM/C scar happens during the resection of the cutaneous and subcutaneous periclitoral scar and is performed with cold blade, scissors or electro surgery.

Women who request this procedure receive multidisciplinary care and follow-up examinations comprising health education about sexual anatomy, physiology, and function, as well as FGM/C and clitoral reconstruction. Detailed information is provided about the surgical technique, its outcomes, and the current lack of conclusive evidence. Women also undergo pre- and post-operative psychosexual therapy with a psychiatrist or psychologist who specializes in sexology. This includes psychotherapy, cognitive-behavioral interventions, systems/couple interventions, and eventual psychodynamic interventions aimed at restoring lasting and satisfying sexual function and sexual health. Past traumatic events other than FGM/C, that could negatively impact sexual health, are also screened and treated. In most cases, information, counseling, and psychosexual therapy is sufficient for the women and only few patients finally go for surgery. Women will undergo clitoral surgery if they suffer from clitoral pain or still consider surgery as a rehabilitation and improvement of body image or female completeness.

We retrospectively reviewed the medical files of all women who attended our specialized clinic for women living with FGM/C and were diagnosed with a traumatic neuroma of the clitoris. We collected socio-demographic, clinical, surgical, and histopathological information. The information included age, original country, age at which FGM/C was performed, reasons for consulting our clinic, long-term complications of FGM/C (e.g., vulvar pain or dyspareunia), sexual function, clinical findings (e.g., FGM/C type), surgical treatment performed (e.g., defibulation, clitoral reconstruction, excision of a painful mass), treatment outcome, and histological findings.

All resected material was fixed in 4% formalin for 24 h, then embedded in paraffin. The tissue was cut into 4-μm-thick sections that were stained with hematoxylin-eosin. In one case we had to perform immunohistochemical staining with an anti-S100 protein antibody to outline the neural structure.

Our study was approved by the Swiss Ethics Committees on Research Involving Humans (Protocol Number 13–133 R). Informed consent for the retrospective review of medical files was waived, because contacting the women would have been difficult because of frequent change of contact details.

At the time of surgery, six out of seven women agreed to sign an informed consent authorizing scientific publication of their anonymized medical images.

## Results

Since 2010, we identified seven women who were diagnosed with neuroma of the clitoris after FGM/C. Six of them underwent clitoral reconstruction. Between January 1, 2013 (the date at which we began performing clitoral reconstruction) and June 30, 2016, a total of 25 women initially requested clitoral surgery. After our multidisciplinary care and counseling, only six (24%) women were still willing to undergo the procedure.

A summary of the socio-demographic, clinical, and histopathological features of the seven women presenting with a post-traumatic neuroma of the clitoris is shown in Table 1. Women were 34.14 ± 3.47 years old on average. Five of the patients came from West African countries and two from Somalia. All had undergone FGM/C in their home country during childhood, between the ages of 1 month and 12 years old.

**Table 1:** Clinical features of the seven cases of clitoral neuroma after FGM/C

Case	Age (years old)	Original country	FGM/C type	Age at moment of FGM/C	Reason for consulting	Treatment	Histological results	Length of follow-up	Outcome
1	25	Somalia	IIIb	9 yearsold	Chronically painful clitoral mass. Not sexually active	Excision of the clitoral mass; defibulation; sexual counseling.	Foreign body granuloma to ancient suture stitch. Post-traumatic neuroma of the clitoris .	>12 months	Resolution of pain. Not sexually active/
2	38	Burkina Faso	IIc	1 month	Chronic clitoral pain. Dyspareunia at the site of FGM/C. Desire to reduce pain, and improve body image and female identity. Request for clitoral reconstruction.	Clitoral reconstruction and psycho-sexual therapy	Post-traumatic neuroma of the clitoris.	>12 months	Resolution of pain. Improved sexual function: body image, gender identity, lubrication, sexual pleasure, arousal, orgasm.
3	38	Ivory Coast	IIIa	During *early* childhood	Dyspareunia at the site of FGM/C. Desire to improve body image and female identity. Request for clitoral reconstruction.	Clitoral reconstruction and psycho-sexual therapy.	Foreign body giant cellular formation. Post-traumatic neuroma of the clitoris.	>12 months	Resolution of pain. Unchanged sexual function.
4	21	Ivory Coast	IIc-IIIa	7 years old	Dyspareunia at the site of FGM/C. Desire to improve body image and female identity. Request for clitoral reconstruction.	Clitoral reconstruction, defibulation. and psycho-sexual therapy.	Foreign body giant cellular formation. Sub-cutaneous post traumatic neuroma of the clitoris of 0.5 cm.	6–12 months	Improved sexual function: body image, gender identity, lubrication, and orgasm.
5	49	Mali	IIIa	6 years old	Request for clitoral reconstruction to improve body image, female identity, and sexual function. Absence of pain. Presence of sexual pleasure and orgasm.	Clitoral reconstruction and psycho-sexual therapy.	Two post-traumatic neuromas of the clitoris: 1 sub-cutaneous of 0.2 cm and 1 cutaneous.	6–12 months	Improved sexual function: body image, gender identity, and sexual pleasure. Unchanged orgasm.
6	34	Somalia	IIIa, previously defibulated	4 years old	Request for clitoral reconstruction to improve body image, female identity, and sexual function. Absence of pain. Presence of sexual pleasure and orgasm.	Clitoral reconstruction and psycho-sexual therapy.	Post-traumatic neuroma of the clitoris of 0.4 cm	>12 months	Improved sexual function: body image, gender identity, lubrication, and orgasm.
7	34	Burkina Faso	IIc	12 years old	Request for clitoral reconstruction to improve body image, female identity, and sexual function. Absence of pain. Presence of sexual pleasure. Absence of orgasm.	Clitoral reconstruction and psycho-sexual therapy.	Two post-traumatic neuromas of the clitoris: 1 sub-cutaneous and 1 cutaneous.	<3 months	Improved body image and gender identity. Sexual intercourse had not yet been resumed.

Four women presented with FGM/C type III (infibulation) according to WHO classification [3], which entailed cutting of the clitoris. Two of the women had FGM/C type II (excision of the labia and clitoris). One had a FGM/C that was classified as II–III. Clitoral pain was present in four out of seven of the women.

**Fig. 1:**
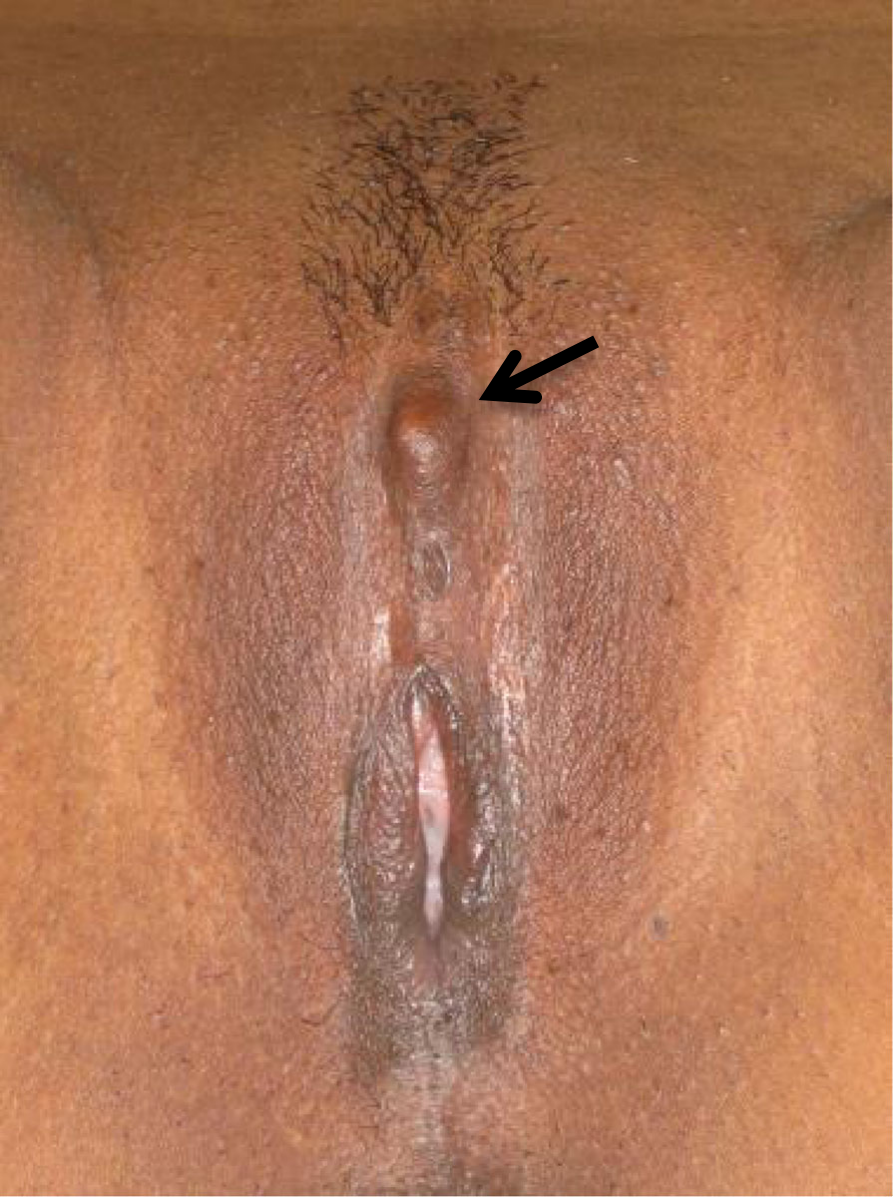
FGM/C type II–III with cutting of the clitoris complicated by a painful clitoral neuroma (*arrow*). Courtesy of JSM

**Fig. 2:**
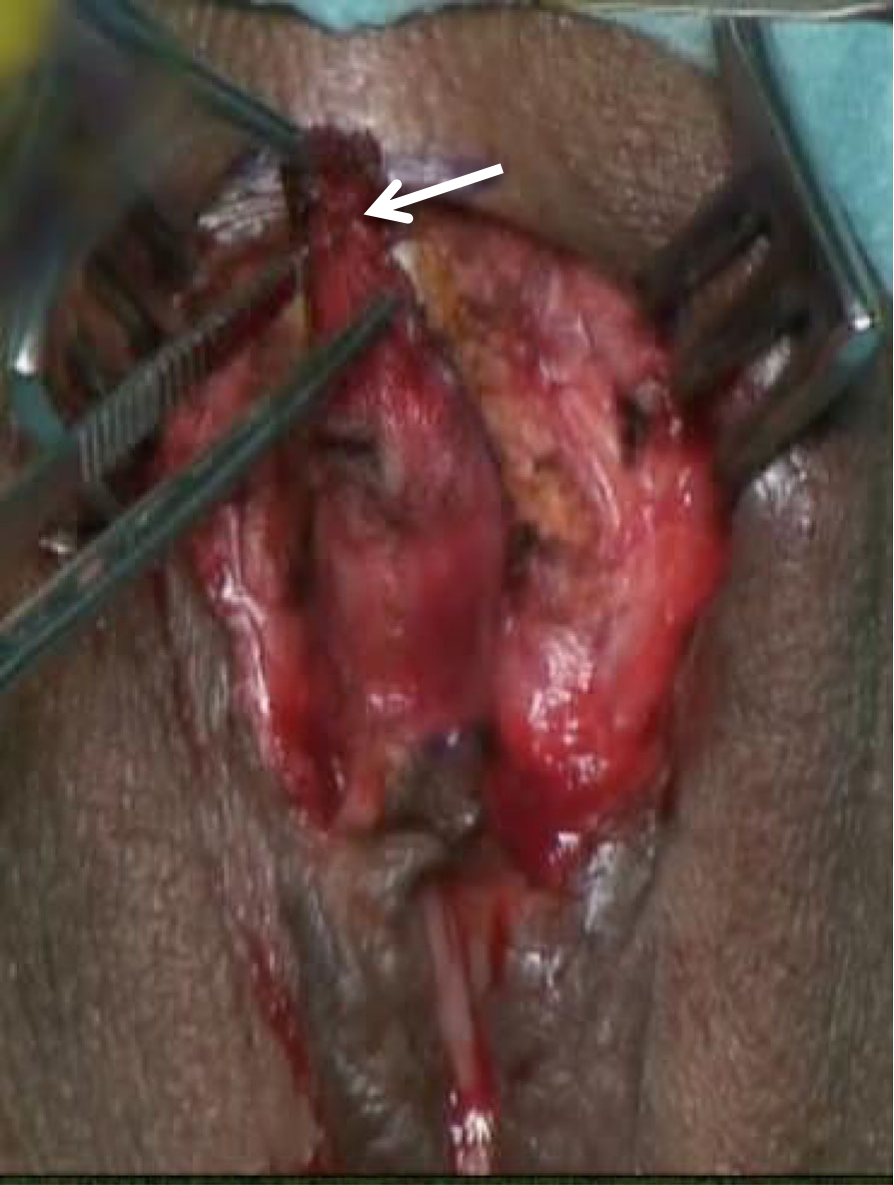
Clitoral reconstruction in FGM/C type II. A post-traumatic neuroma of the clitoris was found within the peri-clitoral fibrotic tissue (*arrow*) removed around the clitoral stump

Only one of the women presented with a visible and palpable painful clitoral mass that was diagnosed as a clitoral neuroma (Fig. 1). Each of the six women who came for clitoral surgery had a FGM/C scar that appeared uncomplicated and was soft and thin upon examination (Fig. 2). Among these, two suffered from dyspareunia in the clitoral region. Pain was evident during intercourse, immediately post-coitus, or both. One woman suffered from chronic clitoral pain (when sitting, touching, washing, and wearing tight clothes) and dyspareunia in the clitoral region. These four women described their pain as severe stinging and burning, as well as painful electric discharges. Their pain adversely affected their sexual lives, both physically and psychologically, and negatively affected their relationships with partners. The remaining three women did not have any vulvar pain. Two of these experienced orgasm and sexual pleasure. One experienced sexual pleasure, but not orgasm.

Of the six women who underwent clitoral reconstruction, three wanted to undergo surgery to improve pain symptoms and sexual function. The other three did not suffer from pain, but considered clitoral reconstruction a way to improve their genital appearance and gender identity, regain something that was removed without their permission, and eventually improve their global sexual function.

The patient who suffered from a painful clitoral mass consulted to have the vulvar pain treated. After receiving information, she was not willing to undergo clitoral reconstruction. She agreed to undergo defibulation at the same time as excision of the mass. By 2 weeks after excision of the clitoral mass, the vulvar pain had ceased.

Women who experienced pain and underwent psychosexual therapy and clitoral reconstruction experienced amelioration of their clitoral pain 3 months post-surgery at the latest – a time period where clitoral neo-glans re-epithelialization was accomplished and post-surgical pain had subsided. Sexual function improved in five of the women. One subject was not sexually active, and one had not yet resumed sexual intercourse (clitoral reconstruction had been performed less than 3 months prior).

Women who asked for clitoral surgery to treat body image and gender identity issues experienced feeling more complete and feminine, as well as improved overall sexual function (Table 1).

**Fig. 3:**
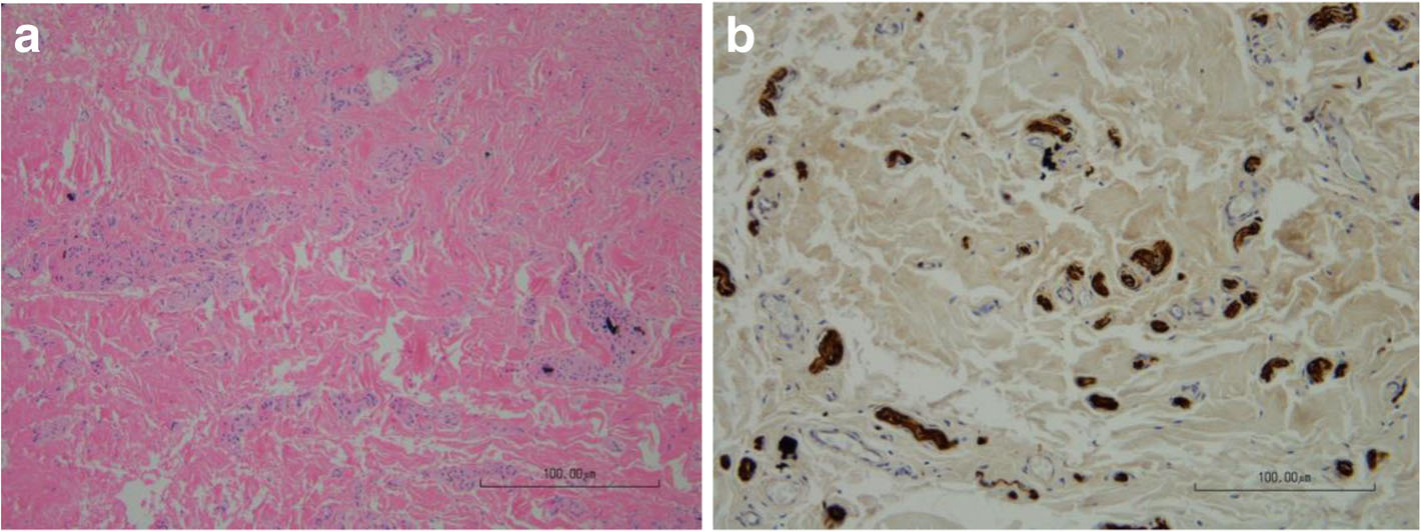
Clitoral neuroma. **a** Hematoxylin-eosin staining shows a disorganized small nerve proliferation in a fibrous tissue. Black dots are remaining foreign material. **b** Anti-protein S100 immunohistochemistry outlines the small nerve structures (magnification 100×)

Histopathological analysis revealed a post-traumatic neuroma of the clitoris in all seven cases (Fig. 3). As shown in Table 1, some subjects presented with a foreign body reaction to previous suture stitches. The number of neuromas did not correlate with the presence of pain symptoms. The short- to medium-term follow-up periods revealed no recurrence or persistency of pain after surgery in our patients.

## Discussion

Our results in this small retrospective study showed that traumatic clitoral neuromas were common findings associated with FGM/C scarring. All women who underwent clitoral reconstruction at our clinic presented with at least a neuroma of the clitoris in the peri-clitoral scar tissue that was removed during surgery, although the neuroma was not always associated with pain or sexual symptoms. Four out of seven women experienced neuropathic clitoral pain.

Pain after nerve injury is called neuropathic pain and is defined by the International Association for the Study of Pain as “pain directly resulting from a lesion or disease affecting the somatosensory system” [9]. Neuropathic pain can persist long after an injury has healed. As our patients experienced, it can include allodynia (pain induced by innocuous stimuli such as touch) and hyperalgesia (severe pain induced by painful stimuli) and it is often described as burning, needling, or electrical-type sensations [10]. Therefore, painful neuromas can severely impact quality of life and cause functional impairment and psychological distress [11]. The psychosexual well-being and daily life of the four women who suffered from clitoral pain were greatly affected. Providers who care for women with FGM/C should consider a diagnosis of clitoral neuroma in patients with neuropathic clitoral pain, even when a palpable or visible painful mass is not visible. Comprehensive multidisciplinary care should include counseling, psychosexual therapy, and surgical treatment.

In our study, women who presented with pain symptoms were treated by surgical excision to remove the clinically visible or invisible neuroma. Clitoral reconstruction envisages excision of the fibrosis surrounding the clitoris, as shown in Fig. 2 [8]. This method allows for the removal of an eventually painful neuroma. To date, clitoral reconstructive surgery has been studied in a mixed population of symptomatic and asymptomatic women [9]. Further studies should separately assess the outcome of clitoral reconstruction in women who suffer from clitoral pain and should perform histology on the excised fibrotic tissue. These results could help to provide a better understanding of the best candidates for this surgical technique, as well as to evaluate its outcomes in terms of efficacy of pain reduction, improved sexual function, and post-operative complications. No evidence exists regarding the risks of clitoral neuromas following clitoral reconstructive surgery. Previous studies have recommended performing the technique close to the pubic symphysis during sectioning of the suspensory ligament of the clitoris before recreating a neo-glans. This avoids damaging the dorsal neurovascular pedicle of the clitoris [12, 13]. Indeed, one study focused on the course of the dorsal nerve of the clitoris (DNC) in six adult female cadavers, showing that the distance of the DNC from the midpoint of the pubic symphysis ranges between 1.6 and 4.8 cm, and no nerves exist at the 12-o’clock position. The DNC is a sensory nerve that divides into two cords at 1 o’clock and 11 o’clock along the body of the clitoris, terminating at a mean distance of 1 cm short of the tip of the glans [14], without reaching the tip of the intact clitoris [14–16].

Chronic clitoral pain greatly impacts relationships, as well as daily and psychosexual experiences, and in our experience, can also recall memories and sensations from the past FGM/C experience. An appropriate psychosexual follow-up should always accompany the surgery to allow better screening and care for additional past traumatic life events (e.g., forced marriage, war, rape) that are often found in migrant and vulnerable populations [17, 18]. The immediate post-operative analgesia should be adequate [17], and surgical and psychosexual follow-ups should be part of the normal routine. Multidisciplinary care includes health education and sex therapy, which allows practitioners to meet the needs of many women without performing clitoral surgery. Our data are similar to previous findings showing that after psychosexual care, only a few women still ask for clitoral reconstruction [17].

One of the limitations of our study was the small sample size, which does not allow for generalization of the results. We also retrospectively reviewed the medical files, but did not use validated scales to assess sexual function. However, the gynecologist who attends, operates, and performs the follow-up examinations on all the women at the clinic reviewed the medical files. Additionally, although validated scales were not used, the medical files were documented with detailed information on sexual function (e.g., body image, gender identity, sexual pleasure, lubrication, pain, and orgasm) independently collected by the surgeon and sex therapist. Further research could focus on the validation and development of questionnaires and scales to assess sexual function among women with FGM/C, to be used also among those subject that undergo surgery.

Our findings are novel and important. First, they improve the available scarce evidence on effective treatments for clitoral pain after FGM/C. Second, they provide input for further studies on clitoral reconstruction outcomes among women suffering from clitoral pain. Our study explains for the first time why clitoral reconstruction can improve clitoral pain symptoms after FGM/C from a pathophysiological point of view. We can conclude that post-traumatic clitoral neuroma is a consequence of FGM/C, and is most likely due to cutting of the dorsal nerve of the clitoris. Nerve resection can cause chronic or sexual clitoral pain or be asymptomatic. Previous work has shown that neuromas of other anatomical sites are symptomatic in 3–5% of cases [19]. The pathophysiological mechanisms of neuroma-associated pain remain poorly understood, and several theories have been proposed, such as fibrous stroma modification, mechanical irritation, or persistent stimulation [20]. Surgical excision has been reported to be a more successful treatment than pharmacological management [19]. In our sample, surgical excision, which was part of a comprehensive multidisciplinary treatment paradigm, provided an effective treatment for clitoral pain due to a post-traumatic neuroma.

Finally, these results provide additional scientific evidence for the possible complications due to FGM/C, increasing the arguments for preventing this practice in future generations.

## Conclusions

Post-traumatic clitoral neuroma can be a consequence of FGM/C. It can cause clitoral pain or be asymptomatic. In the case of pain symptoms, effective treatment is neuroma surgical excision, which can be performed during clitoral reconstruction. Surgery should be considered as part of multidisciplinary care. The efficacy of clitoral reconstruction to treat clitoral pain should be further assessed among symptomatic women.

## Abbreviations

FGM/C: Female genital mutilation/cutting
WHO: Worlds Health Organization
